# Editorial: Voice Technology and Conversational Agents in Health Care Delivery

**DOI:** 10.3389/fpubh.2022.887492

**Published:** 2022-05-30

**Authors:** Emre Sezgin, Shona D'Arcy

**Affiliations:** ^1^NORC at the University of Chicago, Chicago, IL, United States; ^2^Nationwide Children's Hospital, Columbus, OH, United States; ^3^Kids Speech Labs, Dublin, Ireland

**Keywords:** voice technology, chatbot, conversational agent (CA), healthcare, digital health (eHealth)

Conversational agents (CA) are artificial intelligence (AI) driven applications that can process naturalist text and speech to deliver personalized interactions with users, i.e., chatbots, voice assistants, embodied conversational agents, intelligent virtual assistants, dialogue systems, and interactive voice recognition systems (IVRS). The inclusion of automatic speech recognition, natural language processing, and machine learning algorithms help to understand and react to natural conversations and learn from each interaction. Deloitte reported that the global conversational AI market is expected to reach ~US$14 billion by 2025, and chatbots are mostly used AI solutions, the top use of AI in the industry (1). Similarly, the adoption of smart speakers is increasing, and by 2021, ~94 million U.S. adults own at least one smart speaker (2).

The range of applications and demand for CA within healthcare is also growing, for patient-facing and healthcare providers-facing applications. During the pandemic, CA has been highly utilized tools by CDC and WHO to provide COVID19 screening services for populations and add value to be a remote care delivery tool (3). Furthermore, conversational sentiments, vocal attributes, and speech biomarkers are promising fields that will potentially help improve preventative care and remote patient monitoring, enabling early detection of symptoms, and promoting PGHD and PRO (4–7). Overall, CA provides an exciting opportunity to develop new approaches for interactions that can be delivered in a multimodal and naturalistic way to enhance healthcare. Yet, it is important to consider the additional design and deployment requirements when delivering CA solutions for healthcare. The articles submitted to this Research Topic highlight the excellent work being carried out to understand the human interaction required to deliver effective solutions via CA and the challenges that uniquely face healthcare applications.

With the advancement of NLU/NLP, CA has improved over time to understand the language and maintain natural conversations. Palanica and Fossat present a study to compare the comprehension accuracy of intelligent virtual assistants, which is a subset of CA (i.e., voice assistants, namely, Amazon Alexa, Google Assistant, and Apple Siri), to recognize commonly used medication names in the U.S. They replicate their earlier study to observe the changes between 2019 and 2021. Their findings show approximately 10% to 24% increase in performances of Amazon Alexa and Apple Siri. Among all, Google Assistant is found to be the best intelligent virtual assistant that comprehends the brand name and generic name of the medications. However, in comparison to the first study, the authors find slight differences in the performance of Apple Siri and no difference in the performance of Amazon Alexa and Google Assistant. They provide further insight on overall improvement in these intelligent virtual assistants toward becoming more usable in everyday life across different user demographics, emphasizing the value of intelligent virtual assistants in remote healthcare delivery and telemedicine.

The voice interactive CA increases the convenience in care management. Sezgin et al. investigate the feasibility of using voice interaction and automatic speech recognition enabled on smartphones to keep medical notes and track symptoms and medications for the patients. They conduct a pre-post study with caregivers of children with special healthcare needs (pre-*n* = 41, post-*n* = 24) to observe the caregiver preferences before and after using the voice interactive research app (SpeakHealth). The results are descriptively analyzed. The authors report that the majority of the participants kept voice interactive notes <20 s in length focusing on symptoms and conditions, medications, treatment, and therapies, and patient behaviors. The caregivers respond that the voice interaction and transcribed notes positively change their preference of technology to use and methods for tracking symptoms and health events at home. The findings support that voice interactive tools are feasible and effective in remote care management. The authors further discuss the implementations with voice interactive tools, including interoperable and integrated medical records, improving patient-reported outcomes through the rich unstructured patient-generated health data (through transcriptions), and improving speech biomarkers with generated real-world data.

The technology supporting CA and interactive technologies has accelerated significantly in recent years but the user research to ensure engagement, and adoption is still lagging. Ollier et al. investigate the potential impact of components of language on the acceptability of CA. Their study provides insight for CA designers to consider linguacultures when developing healthcare solutions employing CA. The authors examined the Tu/Vous distinction across German and French in a healthcare CA application and found that French speakers prefer a more formal form of address (vous) compared to Germans. The authors additionally report that the term of address can influence users' evaluation outcomes when age and gender are considered. This work is important to demonstrate the underlying lingua-cultural factors that can affect the acceptance of a CA. Deploying CAs in the healthcare setting requires accurate and complete data collection for successful application. This manuscript presents evidence of additional user experience that should be tested when developing CA applications.

Health and wellbeing is the biggest growing market segment in the voice technology market (8). With so many potential applications and ease of deployments through the skills marketplace, it is important to establish a framework for user experience and validating solutions for conversational health agents (CHA). Maharjan et al. investigates 2,741 critical reviews of 485 Amazon Alexa Skills listed under the “health and fitness” category using structural topic modeling technique. They align the top 15 subjects of user critique with a well-established hierarchy of user needs. Mapping commonly found critique subjects of the skills to themes, such as “Functional,” “Reliable,” “Usable,” and “Pleasurable,” helps to identify the important design components related to user experience for CHA. This analysis of user critiques is important for designers of healthcare apps. The authors also highlight the foundational needs of CHA design and outline the pitfalls that designers can avoid for ensuring better engagement and outcomes. The analysis approach is recommended since individual reviews can highlight opportunities for new features, identify bugs, highlight pain points and provide rich insight about the user's emotional experiences and broader social context.

One of the biggest challenges for any healthcare innovation is the behavior change associated with moving away from an attitude of “what we are doing now is good enough.” Grimm et al. identified the importance of a clear strategy to show potential users the added value of adopting new tools. In their study, the authors report the development of a CA for promoting capacity building to enhance the ability of organizations to address health issues and concerns. The scope of their work is toward developing digital formats for the delivery of training and intervention for team leaders. The addition of machine learning to enhance the capabilities (from an “if-then-else” rule-based system to a more intuitive, personalized, and responsive system) is proposed to deliver a scalable and efficient solution. The authors present a step-by-step description of the development of a chatbot from theory to initial prototype and to evaluation with actual users. The authors capture the challenges in trading off initially increased cognitive load with eventual improvements in working conditions. This work illustrates good practices on how to develop solutions by integrating the right users or stakeholders at the right stage of development.

In this Research Topic, the studies present evidence and insight about the implementations of CA toward individual wellbeing and healthy lifestyle, the increasing value of data collected through CA in remote care management and patient-generated health data, the value of user experience and design of CA, and improvements and challenges on natural language understanding and linguistic approaches (Figure 1).

**Figure 1:**
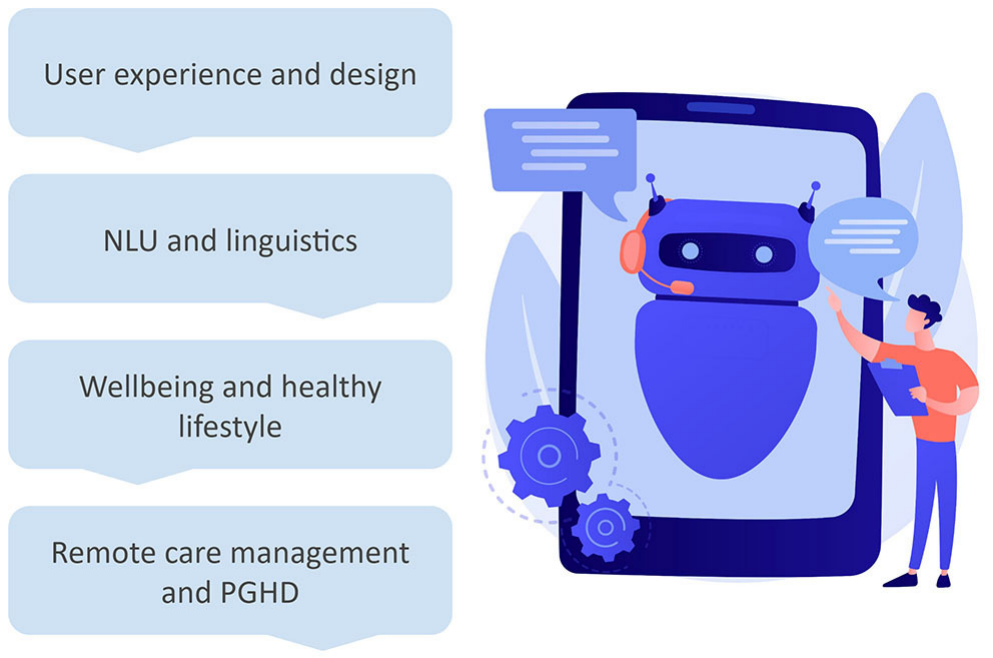
Special issue's content topics at a glance. NLU, natural language understanding; PGHD, patient-generated health data. The figure has been designed using an image from Freepik.com.

## Author Contributions

All authors listed have made a substantial, direct, and intellectual contribution to the work and approved it for publication.

## Conflict of Interest

The authors declare that the research was conducted in the absence of any commercial or financial relationships that could be construed as a potential conflict of interest.

## Publisher's Note

All claims expressed in this article are solely those of the authors and do not necessarily represent those of their affiliated organizations, or those of the publisher, the editors and the reviewers. Any product that may be evaluated in this article, or claim that may be made by its manufacturer, is not guaranteed or endorsed by the publisher.
